# The unusual and dynamic character of PX-DNA

**DOI:** 10.1093/nar/gkv739

**Published:** 2015-07-15

**Authors:** Dong Niu, Hualin Jiang, Ruojie Sha, James W. Canary, Nadrian C. Seeman

**Affiliations:** Department of Chemistry, New York University, New York, NY 10003, USA

## Abstract

PX-DNA is a four-stranded DNA structure that has been implicated in the recognition of homology, either continuously, or in an every-other-half-turn fashion. Some of the structural features of the molecule have been noted previously, but the structure requires further characterization. Here, we report atomic force microscopic characterization of PX molecules that contain periodically placed biotin groups, enabling the molecule to be labeled by streptavidin molecules at these sites. In comparison with conventional double stranded DNA and with antiparallel DNA double crossover molecules, it is clear that PX-DNA is a more dynamic structure. Furthermore, the spacing between the nucleotide pairs along the helix axis is shorter, suggesting a mixed B/A structure. Circular dichroism spectroscopy indicates unusual features in the PX molecule that are absent in both the molecules to which it is compared.

## INTRODUCTION

**Figure 1.**
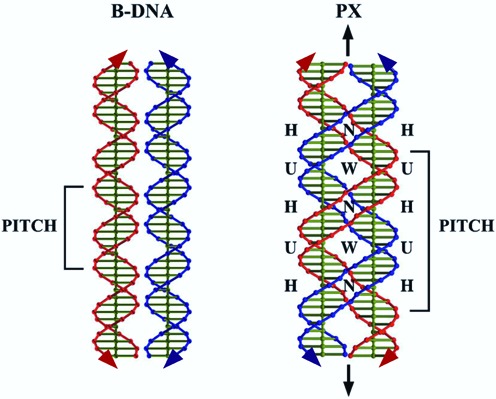
Comparison of B-DNA and PX-DNA. Two B-DNA double helices are shown on the left and PX-DNA is shown on the right. Other than B-DNA, PX-DNA appears to consist of inter-wrapped blue and red double helices. The dyad axis of PX-DNA is indicated as black arrowheads above and below the drawing. For any given half-turn of DNA, either a major (wide) groove separation (indicated by **W**) or a minor (narrow) groove separation (indicated by **N**) will face the dyad axis. The helical pitches of both structures are indicated. Half turns labeled **U** do not require complementarity between the blue and red strands for the PX structure to form, but those labeled **H** do require it, so the structures must be homologous in those regions (2).

PX-DNA is a four-stranded DNA structure in which two double helices appear to wrap around each other with their local helix axes parallel (Figure 1) (1). There are a number of ways to represent the relationships of the individual strands, but the representation shown in Figure 1 emphasizes that two homologous DNA duplexes can interact with each other to form the motif. The most prominent feature of the structure is that crossovers occur at every possible position where the strands approach each other. There is an apparent dyad axis vertical in the plane of the page, representing the spatial relationship expected between the blue strands and the red strands. The resulting structure contains two parallel fused double helices (each a mixture of red and blue strands) that flank the dyad and are related by it. If one examines the left of these flanking double helices, from top to bottom, one sees a red–red half-turn, a red–blue half-turn, a blue–blue half-turn and a blue–red half-turn, before the pattern repeats; the right helix is the opposite, starting with a blue–blue half-turn. Note that the expression ‘half-turn’ is an approximation, because alternating half-turns represent major groove (W, for wide) and minor groove (N, for narrow) spacings. The nature of this repeat bears on the possible role of PX-DNA in the recognition of homology (2): it does not matter whether homology exists in the red–red or the blue–blue domains, only in the half-turns containing red–blue and blue–red hybridization require homology. This type of interrupted homology is known as PX-homology (2).

Recently, PX-DNA has been implicated as a motif that is capable of relaxing superhelical DNA (2), just as cruciforms (3), Z-DNA (4) and H-DNA (5) can. The formation of PX-DNA leads to a structure wherein both the twist and the writhe are decreased within the PX-DNA region (2). Cruciforms relax superhelical DNA when inverted repeats are present, Z-DNA relaxes supercoils by the formation of left-handed DNA in Z-forming sequences and H-DNA is a relaxation motif formed in the presence of mirror-symmetric poly-purine sequences. Similarly, homology-based relaxation requires the presence of homology, either conventional complete homology or PX-homology. The evidence supporting the ability of homologous or PX-homologous sequences to relax DNA supercoils include 2D gel electrophoresis, atomic force microscopic (AFM) data and both *in vitro* and *in vivo* psoralen crosslinking (2). The AFM data demonstrate a shaft-like structure in the region of homology, and although the shaft is thought to be PX-DNA, that has not been demonstrated unambiguously by structural methods. The DNA psoralen-crosslinking data suggest that strands in the two regions of homology must interact with each other. Although PX-DNA has been subject to physical characterization, that characterization is incomplete.

Here, we extend the structural characterization of PX-DNA via *in vitro* experiments that can be performed in model systems. We have contrasted the structural behavior of PX-DNA with the behavior of antiparallel DNA double crossover (DX) molecules (6) and with conventional double stranded B-DNA (DS). Our key structural assay here has entailed the attachment of biotin groups to large DNA constructions and then to examine the positions of streptavidin molecules bound to the biotin groups (7–9) in the AFM. As a function of the positioning of the biotin groups, either linear or zigzag patterns should arise from the presence of the streptavidin molecules. This is reliably the case with duplex (DS) DNA and with DX-DNA (6). Surprisingly, we do not obtain a consistent pattern from PX-DNA, suggesting that relative to the DS and DX molecules its structure is dynamic, and that other methods will be required to establish the detailed character of the homology-based supercoil-relaxing structure. Nevertheless, we do find that AFM characterization of these molecules in this fashion leads to the conclusion that the inter-nucleotide spacings in DS- and DX-DNA are consistent with conventional B-DNA; by contrast, the spacings in PX-DNA suggest a B/A-like structure (10). The structural relationship between the CD spectrum and the structure itself is only qualitative, but it is still instructive to compare the CD spectra of the model systems we have prepared. The CD spectra suggest an unusual structure, roughly in the B-family, but not something observed previously.

## MATERIALS AND METHODS

### Preparation, purification and quantitation of DNA oligonucleotides

Unmodified DNA oligonucleotides were purchased from Integrated DNA Technologies,Inc. (Coralville, IA, USA) and purified by denaturing polyacrylamide gel electrophoresis (PAGE). Biotinylated strands were synthesized using an Applied Biosystems 394 synthesizer with biotin-dT phosphoramidites, purchased from Glen Research, Inc. (Sterling, VA, USA) using conventional phosphoramidite synthetic methods (11) and purified by denaturing PAGE. The deprotection and purification processes were the same as those for the unmodified strands. Concentrations of the DNA strands were estimated by absorption at 260 nm. Sequences for all oligonucleotides are shown in Supplementary Table S1.

### Ligation reaction for preparing long strands

A total of 500 pmole of each of the three short DNA segments (90-mers) and 1000 pmole of each of the two DNA linkers (30-mers) were first mixed in 1× T4 ligase buffer containing 50 mM Tris–HCl (pH 7.5), 10 mM MgCl_2_, 1 mM adenosine triphosphate and 10 mM Dithiothreitol (DTT). The mixture was then heated up to 90°C and gradually cooled to room temperature over 2 h to facilitate hybridization. Twenty units of T4 ligase were then added to the annealed samples. The final mixture was incubated at 16°C for 16 h. The ligated products were purified by denaturing PAGE.

### Formation of the DNA structures

The strands required for each structure were mixed stoichiometrically in 1× TAE/Mg^2+^ buffer consisting of 40 mM Tris–HCl (pH 8.0), 20 mM, acetic acid, 2 mM ethylenediaminetetraacetic acid (EDTA) and 12.5 mM magnesium acetate. The mixture was then heated to 90°C and gradually cooled to room temperature over 48 h. Non-denaturing PAGE gels showing successful formation of DX and PX molecules are shown in Supplementary Figure S1.

### Denaturing polyacrylamide gel electrophoresis (PAGE)

Denaturing gels were cast in 1× TBE buffer, which contained 10–20% (as required) acrylamide (acrylamide:bisacrylamide::19:1) and 8.3 M urea. The samples were dissolved in a loading buffer containing 10 mM NaOH, 1 mM EDTA and a trace amount of Bromophenol Blue and Xylene Cyanol FF. The gels were run on a Hoefer SE-600 electrophoresis unit at 31 V/cm constant voltage at 55°C with 1× TBE as the running buffer.

### Non-denaturing polyacrylamide gel electrophoresis (PAGE)

Non-denaturing gels containing 6% acrylamide (acrylamide:bisacrylamide::19:1) were cast in 1× TAE/Mg^2+^ buffer. One volume of the loading buffer containing 1× TAE/Mg^2+^ buffer, 50% glycerol and trace amounts of Bromophenol Blue and Xylene Cyanol FF were added to the annealed sample solutions. The gels were run on a Hoefer SE-600 electrophoresis unit at 16 V/cm (constant voltage) at room temperature with 1× TAE/Mg^2+^ as the running buffer.

### Preparation of the biotin-streptavidin conjugate labeled DNA structures

Each of the annealed structures was mixed with streptavidin molecules in a molar ratio of 1:10. The mixture was then incubated at 25°C for 2 h to facilitate the binding reaction.

### Atomic force microscope (AFM) visualization

AFM imaging was conducted in air using tapping mode. To deposit the sample, 5 μl of the sample was dropped on a freshly cleaved mica surface for 2 min. Additional 1× TAE/Mg^2+^ was added, as appropriate, to promote deposition. The surface was then washed five times, using 50 μl of double distilled water to remove unreacted streptavidin particles. The mica plate was then dried using an air duster and observed by a Multimode AFM scanner with a NanoScope IV controller.

## RESULTS AND DISCUSSION

We have used a model system to ask whether biotin-streptavidin conjugation is a feasible strategy to characterize the apparently-PX shaft-like structure formed in plasmids containing homology. A group of DNA structures with almost identical sizes, including DNA duplex (DS), double crossover (DX) DNA (6) and PX-DNA (1) have been designed and constructed. They have been used to test whether biotin-streptavidin conjugates can provide structural data on the shaft-like structures formed within homology-relaxed plasmids. A variety of PX-DNA molecules have been described (1,2), but those formed by four separate strands form most successfully with a minor groove spacing of 5 nt pairs, and major groove spacings of 6, 7 and 8 nt pairs (1). In the work described here, we have used a PX molecule designed to contain 6 nt pairs in the major groove and 5 nt pairs in the minor groove. Thus, if the structure were to form as designed, with biotin labels separated by 55 nt, streptavidin units ought to be organized in a zigzag shape.

**Figure 2.**
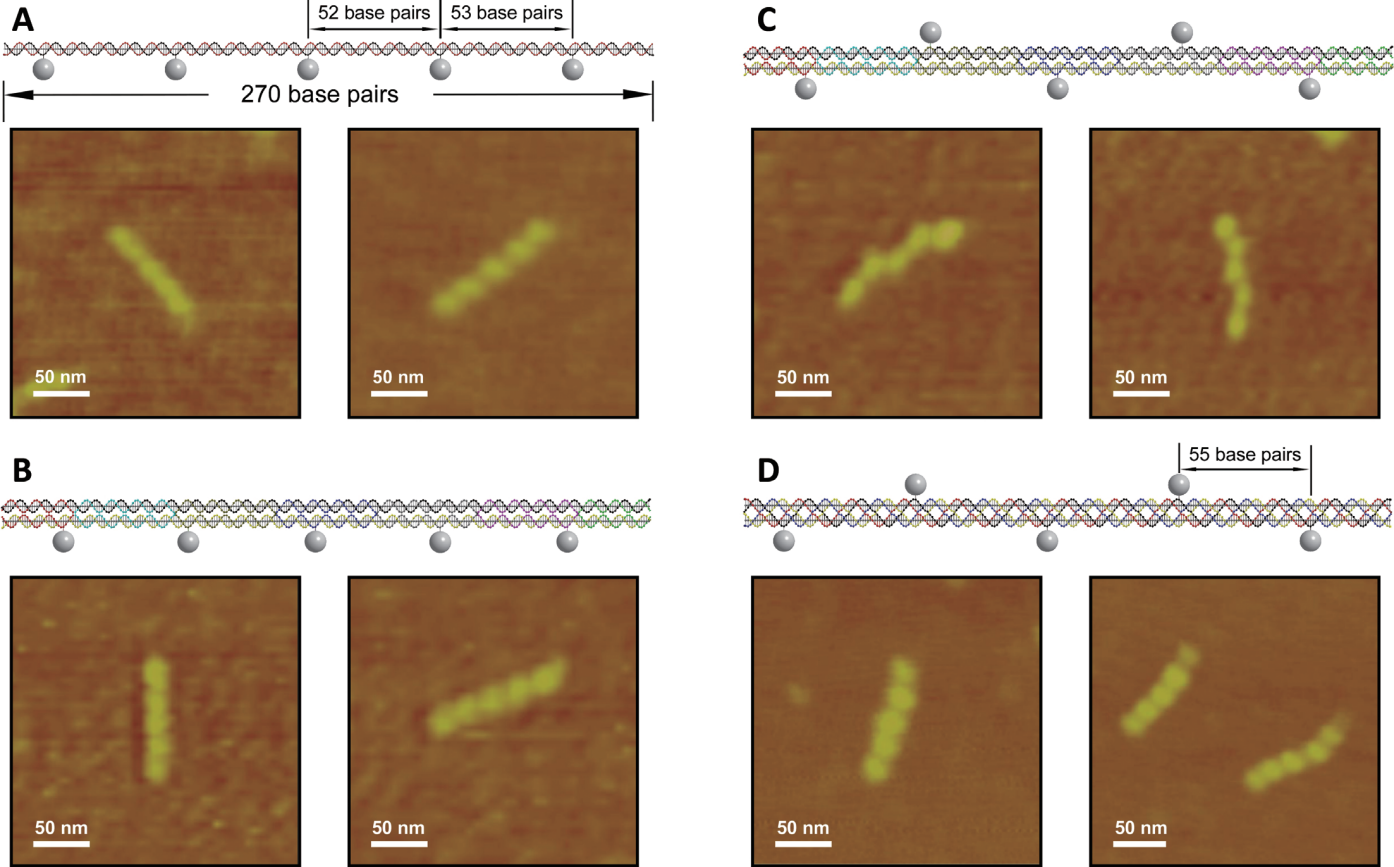
Labeled DNA structures and their corresponding atomic force microscopy (AFM) images. All the characterized DNA structures are 270 nt pairs in length and the separation between adjacent biotin-streptavidin conjugates is 52 or 53 nt pairs in the DS and DX variants, whereas that of the PX variant is 55 nt pairs. (**A**) Linear labeled DS-DNA. (**B**) Linear labeled DX-DNA structure. (**C**) Zigzag labeled DX-DNA structure. (**D**) Zigzag labeled PX-DNA structure.

The first structure we examined was a DNA duplex, expected to assume the B-conformation. For satisfactory visualization resolution, the length of the duplex is 270 bp, roughly 92 nm (12). One strand is labeled with five biotin-streptavidin conjugates with alternate separations of 52 and 53 nt pairs between any two adjacent labels, so as to have all the labels positioned on the same side of the duplex (Figure 2A). The sample was then examined by AFM, and as shown in Figure 2A, the corresponding linear conformation was observed clearly with a frequency of 91%. Zoomed-out AFM images of relevant species are shown in Supplementary Figure S2 to confirm frequencies of occurrence.

The second experiment was conducted on an antiparallel double crossover (DX) DNA construct. The DX-DNA molecule used is a structure composed of two helical domains (6), which bears some similarity to PX-DNA. However, the two helical domains in the DX molecule are joined by only six crossovers over the entire length of the molecule. In addition, the crossovers in this molecule are formed between strands of opposite polarity, rather than strands of the same polarity, which is the case in PX-DNA. The DX molecule is expected to adopt the B-form (13) and to act as a B-form standard, as demonstrated by the CD experiments reported below. Here we made a DX-DNA structure with the same length as the duplex (270 nt pairs). Antiparallel DX-DNA molecules with an integral number of helical turns between crossovers, termed DAE (6), were used. The number of biotins and of biotin-streptavidin conjugates is also five, and the separation between two adjacent labels is also 52 or 53 nt pairs, the same as the duplex structure. DX-DNA was first labeled in a linear arrangement, similar to the duplex above, as shown in Figure 2B. AFM results (Figure 2B) demonstrate that the organization of the labels is indeed linear with a frequency of 92%.

The DX structure was studied further by using a labeling scheme expected to produce a zigzag shape. To implement the zigzag, the second and the fourth labels are switched to the other side of the structure without being moved along the backbone, as illustrated in Figure 2C. The zigzag labeled DX-DNA was clearly observed in the AFM images with a frequency of 80%; in Figure 2C the differences between the linear and the zigzag images are clear. This number suggests that most of the zigzag labeled DX molecules produce the corresponding zigzag pattern. These data are important in two regards: (i) biotin-streptavidin conjugation has been shown to be a viable probe for visualizing the modal conformation of DNA structures; and (ii) if the PX structure had a conformation that could organize biotin-streptavidin conjugates into a zigzag shape, the zigzag would be visible under AFM.

To study the structure of PX-DNA, 6:5 PX (1) was used because half of its 22-nt-pair helical pitch, 11 nt pairs, is close to that of conventional B-form DNA (10.5 bp) (14–15). It is assumed that the similarity in terms of conformation would make it easier to predict the actual structure of PX-DNA. We have designed our sequences so as to guarantee that the actual structure formed is PX-DNA (1). Biotin-streptavidin conjugates are positioned at the same positions as those of the labeled DX-DNA structure above. Owing to the nature of the PX structure, all the labels are on the same strand of the structure, as illustrated in Figure 2D. However, under AFM visualization, we could only see linear arrangements from the labeled PX-DNA structure in Figure 2D. This result is quite different from the zigzag DX-DNA but it is important to recall that our design for PX-DNA was based on B-form DNA, and this is not necessarily the case for the PX structure.

**Figure 3.**
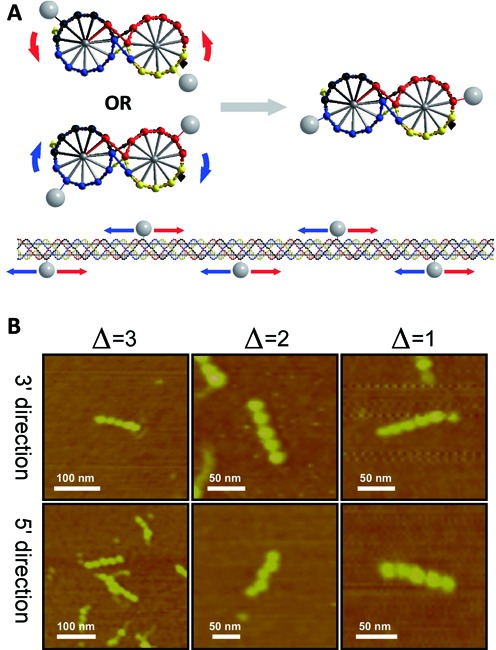
Angular titration experiments to establish the best positions to place the labels. (**A**) Schemes of moving the labels on the zigzag PX-DNA: top: side view; bottom: front view (red arrow: 3′ direction, blue arrow: 5′ direction). (**B**) A matrix of the selected AFM images from each of the variants in the titration experiments.

If the structure of PX-DNA were inconsistent with the B-form DNA conformation, the labeling sites chosen above might not be optimal. One solution to this problem is moving all the labels in either the 3′ or 5′ direction by a fixed small number of nucleotides so as to rotate them by a few nucleotide twist intervals. The rotation is expected eventually to place the conjugates at sites where a clear zigzag could form. Figure 3A illustrates the idea behind this experiment. Considering the rotation per base pair in B-form DNA is roughly equal to 34–36°, moving the labels by a maximum of 3 nt should be sufficient to titrate the required angular range. We have made and tested six different systems, and the AFM results are summarized in Figure 3B. Among all the results, only moving the labels by 2 nt in the 5′ direction sometimes generates a zigzag PX-DNA pattern (32%), whereas all the other five variants largely produce linear PX-DNA streptavidin patterns. Although a small number of barely zigzag molecules were also observed from each of the other variants, the percentages of the absolutely linear patterns from them are all higher than 90%.

**Figure 4.**
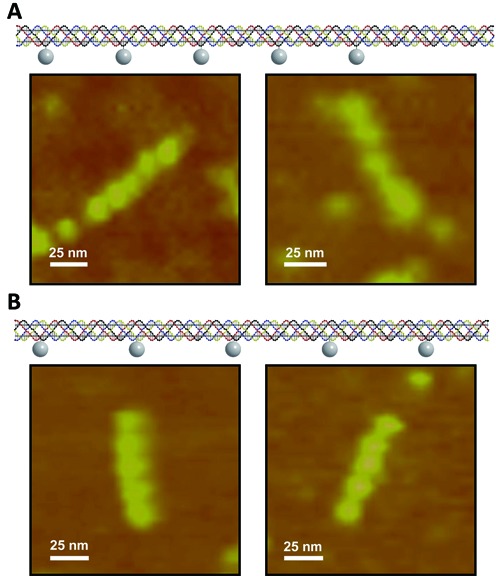
Linear labeled PX-DNA molecules. (**A**) Linear labeled PX-DNA separated by 44 nt pairs and its AFM images. (**B**) Linear labeled PX-DNA separated by 55 nt pairs and its AFM images.

Since most of the angular titration experiments generate linear conformations from the labeled PX-DNA structures, it is necessary to make a PX-DNA control designed to have an exact linear conformation to mark the difference. Thus, we designed another labeled PX-DNA with a separation of 44 nt pairs between any two adjacent conjugates (Figure 4A). Whereas 44 nt pairs is an integral multiple of the helical pitch of 6:5 PX-DNA, all the labels are positioned at the same side of the structure to form a linear control. Figure 4A shows the AFM images from this experiment. The designed linear conformation was clearly observed and there is no obvious conformational difference between this designed linear system and the linear results obtained in the titration experiments on the zigzag labeled PX structures. Another control experiment was conducted by using 55 nt pairs as a separation to label the PX-DNA linearly. Other than the case above, multiple PX strands need to be labeled in this experiment so as to position all the labels at the same side of the structure to yield a linear pattern. As shown in Figure 4B, the linear conformation was also clearly observed without being different from the linear molecules observed previously.

**Figure 5.**
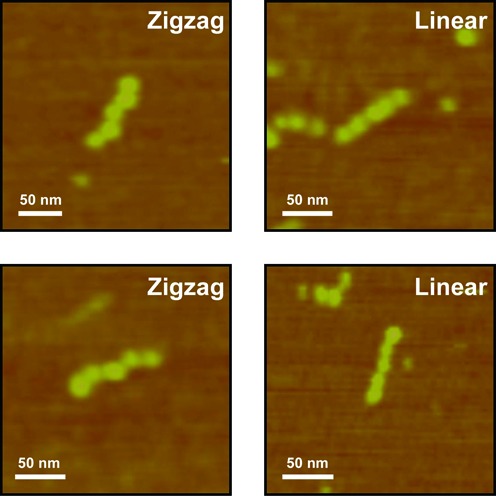
Selected AFM images of the molecules from the variant with 2 nt moved in the 5′ direction. Both zigzag and linear conformations were observed. The linear conformation observed appears not to be different from the linear pattern seen in other cases.

In all of the experimental data, only the titration of moving all the labels 2 nt in the 5′ direction gave a fairly clear zigzag conformation, so we tested more molecules with this modification. However, this modification does not reliably produce the zigzag pattern. As shown in Figure 5, this variant is capable of producing linear molecules as well. Thus, PX-DNA appears to be more dynamic than the DX molecule, despite having more crossovers, which are expected to stabilize the stiffness of the structure (16). We recognize that binding to the mica surface may perturb bound molecules, but the susceptibility of PX-DNA is much greater than DS and DX-DNA, which ought to be subject to the same forces. Molecular dynamics calculations by the Goddard group have previously indicated that the structures of the PX molecule (17) and its close topological relatives (18) are certainly susceptible to fluctuations.

We have noted the following features in the data: first, DNA duplex and DX-DNA, as regular B-form DNA, can produce designed conformations with biotin-streptavidin conjugates.. By contrast, PX-DNA can not organize the labels into a zigzag shape if one assumes that its structure is B-form DNA. This observation suggests that the actual conformation of PX-DNA is not simple B-form DNA. Owing to the result that both the zigzag and linear conformations were observed by AFM in the labeled PX molecules, it suggests that PX molecules assume multiple conformations to a greater extent than in the DX molecule.

We have analyzed multiple images of the streptavidin-labeled DS, DX and PX molecules to estimate the distances between streptavidin labels. The results are listed in Table 1. They reinforce the qualitative observations noted above, that DS and DX molecules are consistent with the parameters of classical B-form DNA (12), with a nucleotide separation of ∼3.4 Å. By contrast, PX-DNA is quite different, with an average spacing of 3.17 Å. The inter-nucleotide spacings noted are consistent with the DNA structures noted by Ho *et* *al*. (10) as B/A structures.

**Table 1.** Separation numbers and helicity data in the DNA structures studied

Structure	Total separation (base pairs)	Total horizontal length (nm)	Average rise per nucleotide (Å)
DS-linear	210	72.3 ± 0.7	3.44 ± 0.03
DX-linear	210	71.6 ± 0.3	3.41 ± 0.02
DX-zigzag	210	72.3 ± 1.0	3.44 ± 0.05
PX-linear	176	55.6 ± 1.2	3.16 ± 0.07
PX-linear	220	69.6 ± 0.6	3.17 ± 0.03
PX (5′, *Δ* = 2) zigzag^a^	220	69.8 ± 0.9	3.17 ± 0.04
PX (5′, *Δ* = 2) linear^a^	220	70.5 ± 1.0	3.20 ± 0.05
PX (5′, *Δ* = 1)^a^	220	70.1 ± 0.4	3.19 ± 0.02
PX (3′, *Δ* = 1)^a^	220	70.4 ± 0.3	3.20 ± 0.01
PX (3′, *Δ* = 2)^a^	220	70.2 ± 0.5	3.19 ± 0.02
PX (5′, *Δ* = 3)^a^	220	70.1 ± 0.3	3.19 ± 0.02
PX (3′, *Δ* = 3)^a^	220	69.7 ± 0.5	3.17 ± 0.03

^a^Variants from the angular titration experiments.

**Figure 6.**
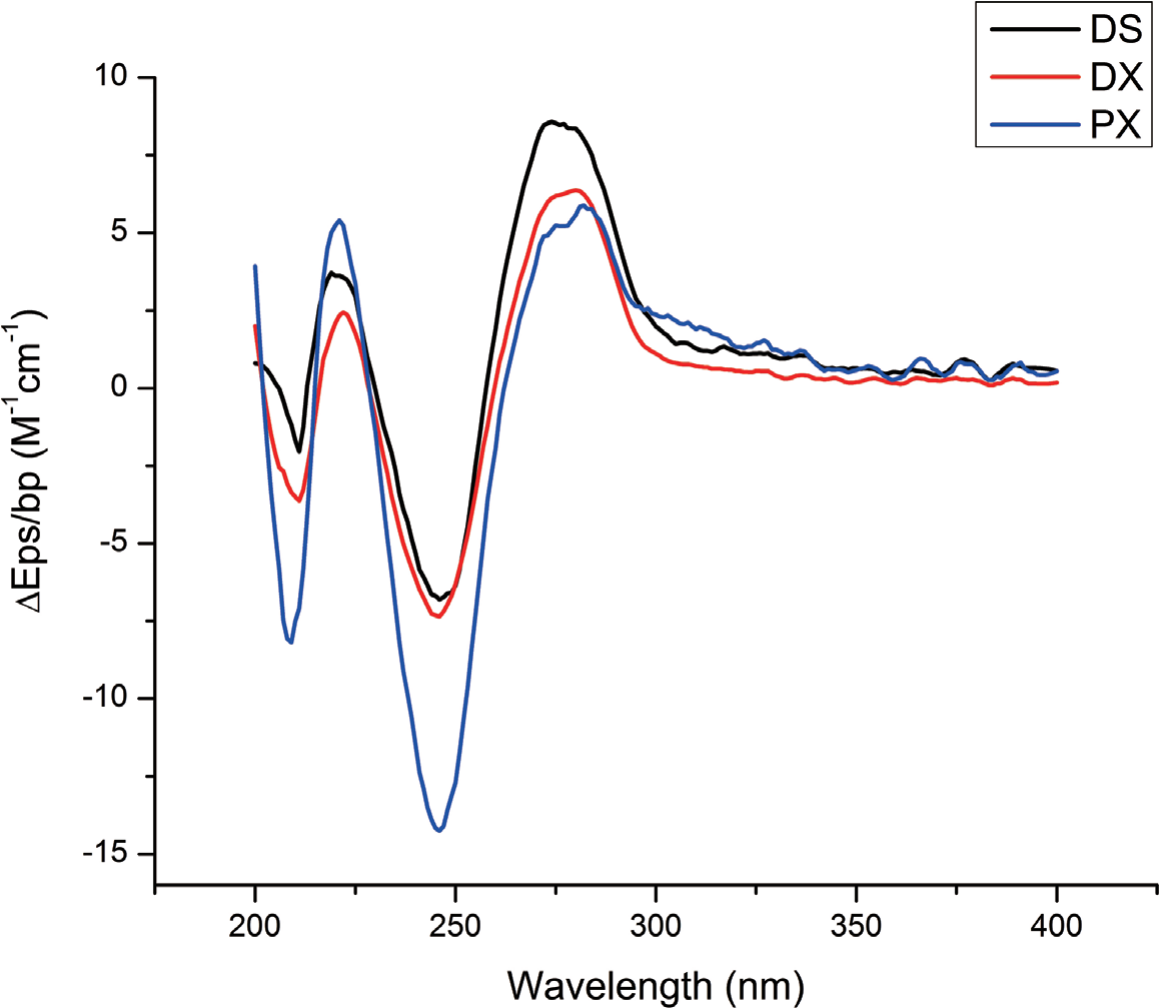
Circular dichroism (CD) spectra of the DNA structures studied. DS and DX-DNA produce spectra corresponding to that of B-form DNA, whereas PX-DNA shows a B-like spectrum but it is clearly not a simple B-like DNA structure.

To gain insight into the nature of these structures, we have performed circular dichroism experiments on the three sets of molecules. The results are shown in Figure 6. They show that DS- and DX-DNA clearly adopt the B-form in our conditions. By contrast, PX-DNA has a B-like spectrum, but it is clearly not a simple B-like spectrum. There was a possibility that with an 11-fold repeat, PX-DNA would appear A-like, but the CD spectrum clearly excludes this possibility. We have analyzed the spectrum for the possibility that it is a mix of 10.4- and 10.2-fold DNA (19), but find that we cannot reproduce the nature of the curve as a linear combination of these two structures.

## CONCLUSIONS

We have designed a group of biotin-streptavidin conjugate-labeled DNA structures and have visualized their conformations in an attempt to develop probes for the PX-DNA structure. DS-DNA and DX-DNA behave as expected and appear to assume the well-known B conformation. Labeling PX-DNA based on our simplest structural hypothesis does not yield a zigzag shape as predicted, implying that the actual conformation of the helical domains of PX-DNA is not the same as that of B-DNA. The titration experiments show that moving the labels in the 5′ direction by 2 nt sometimes produces zigzag structures, but linear molecules are also found in these samples. This suggests a structural variability in PX-DNA, capable of yielding two or more different conformations. The lack of unique conformations for PX-DNA suggests that it would be inadvisable to apply the biotin-streptavidin strategy to establish the PX character of the shafts of homology relaxed plasmids (2). Further characterization methods are needed to establish the nature of the relaxation structures. PX-DNA remains a special and somewhat elusive DNA structure.

## Supplementary Material

SUPPLEMENTARY DATA
