# Supplementary material for: The unusual and dynamic character of PX-DNA
Source: Nucleic Acids Res. 2015 Jul 15;43(15):7201–6. doi: 10.1093/nar/gkv739 (PMC4551946; doi:10.1093/nar/gkv739)
Supplement: SUPPLEMENTARY DATA [file supp_43_15_7201__index.html]

The unusual and dynamic character of PX-DNA — The unusual and dynamic character of PX-DNA — SUPPLEMENTARY DATA 

# The unusual and dynamic character of PX-DNA

## SUPPLEMENTARY DATA

- SUPPLEMENTARY DATA
