# Supplementary material for: The unusual and dynamic character of PX-DNA
Source: Nucleic Acids Res. 2015 Jul 15;43(15):7201–6. doi: 10.1093/nar/gkv739 (PMC4551946; doi:10.1093/nar/gkv739)
Supplement: SUPPLEMENTARY DATA [file supp_gkv739_nar-01281-f-2015-File008.docx]

**Supplementary Data**

**Structure formation:** Non-denaturing PAGE (6% acrylamide) analysis has been conducted to monitor the formation of the DX (1) and PX-DNA (2). As the results shown in Figure S1, both of the structures can form a neat band proving the formation of each structure.

**
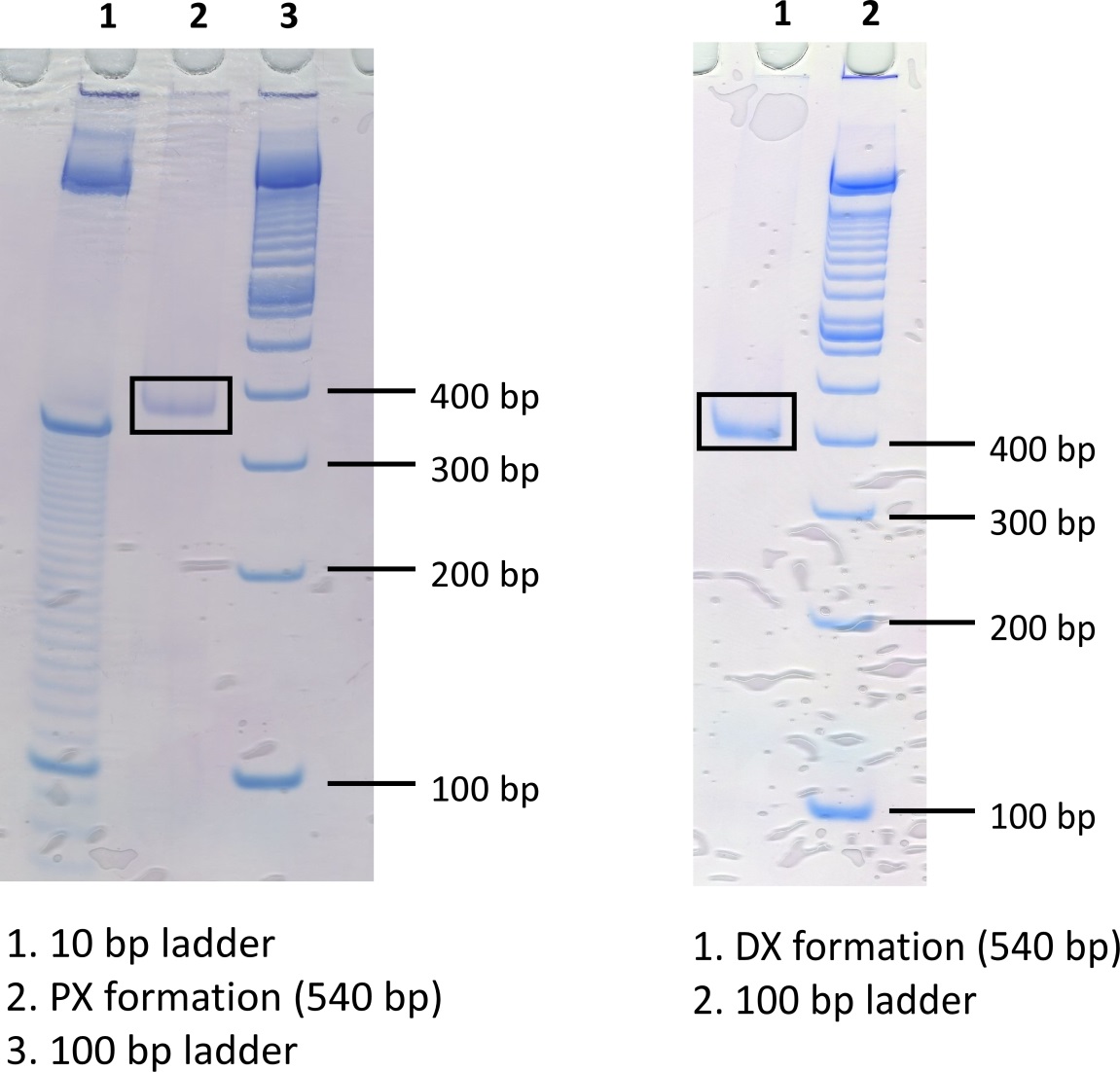
**

**Figure S1.** Non-denaturing PAGE analysis for characterizing the structure formation (left: PX, right: DX)

**
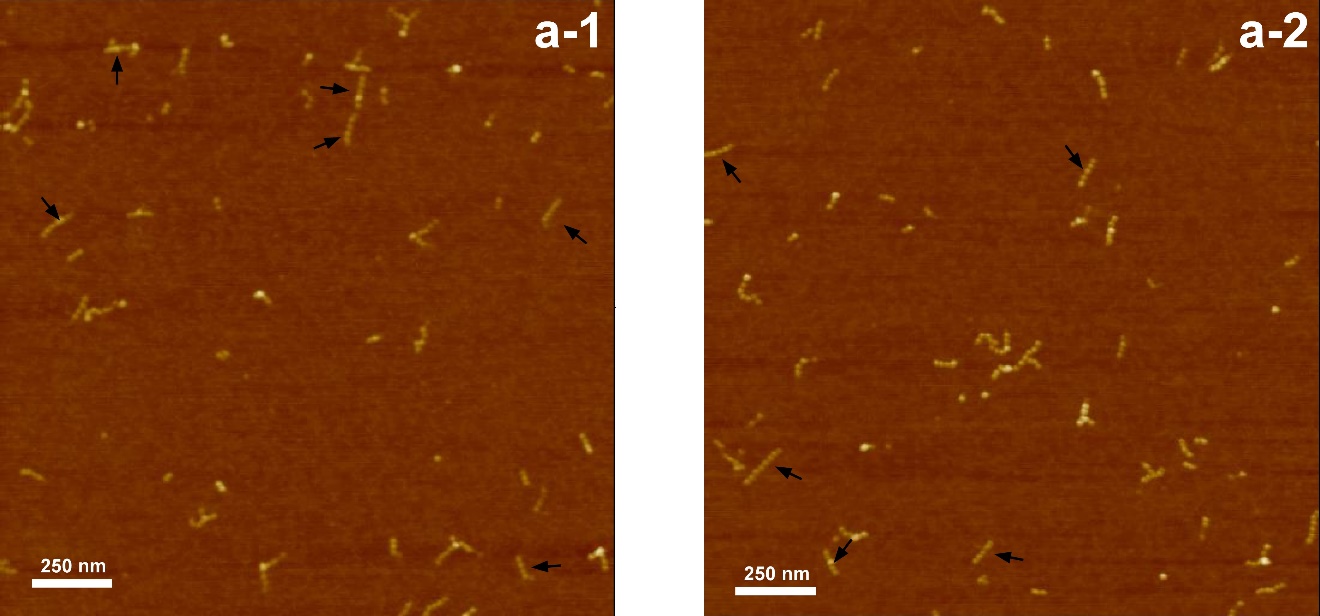
**

**
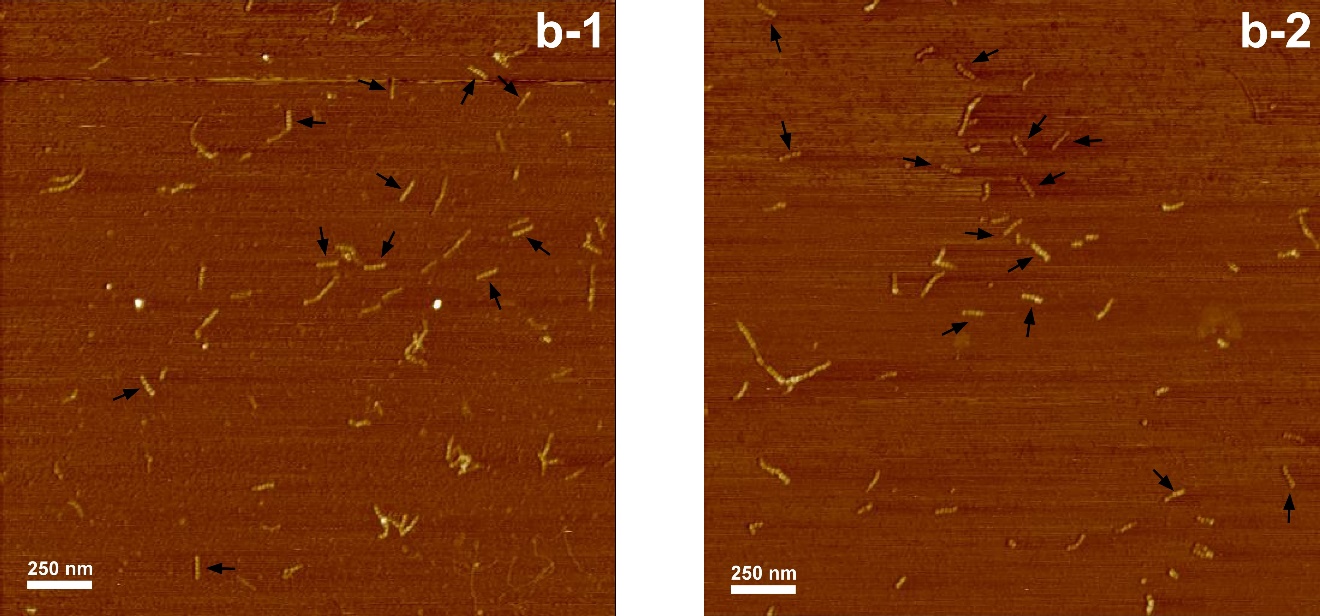
**

**
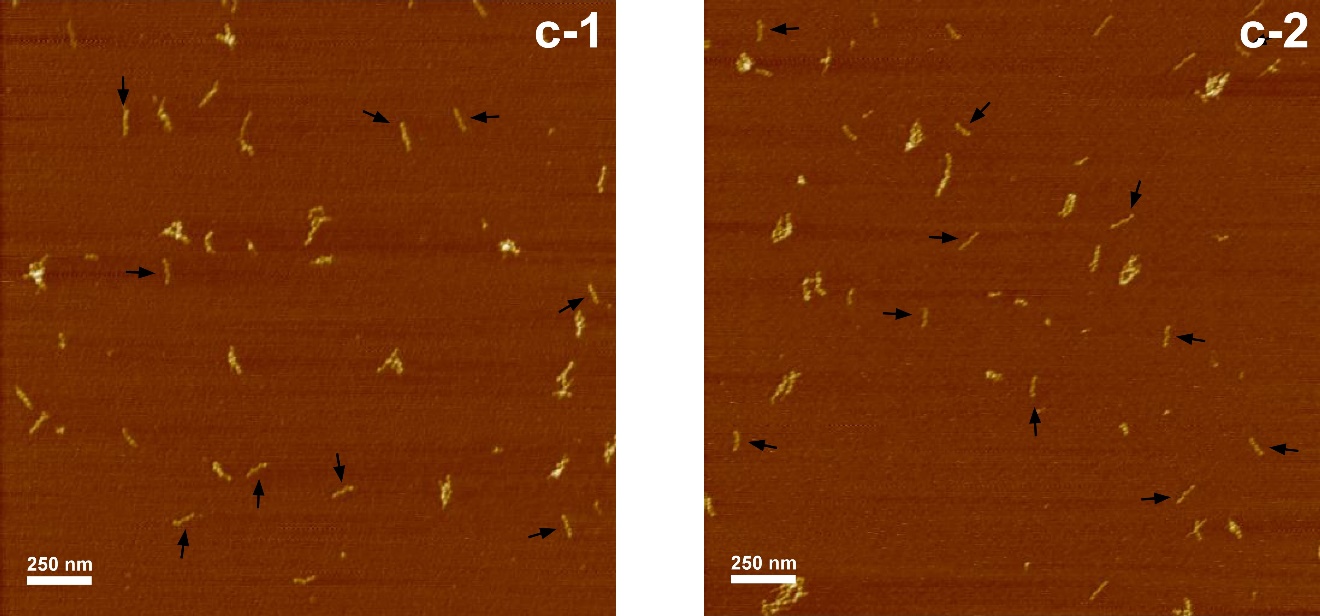
**

**
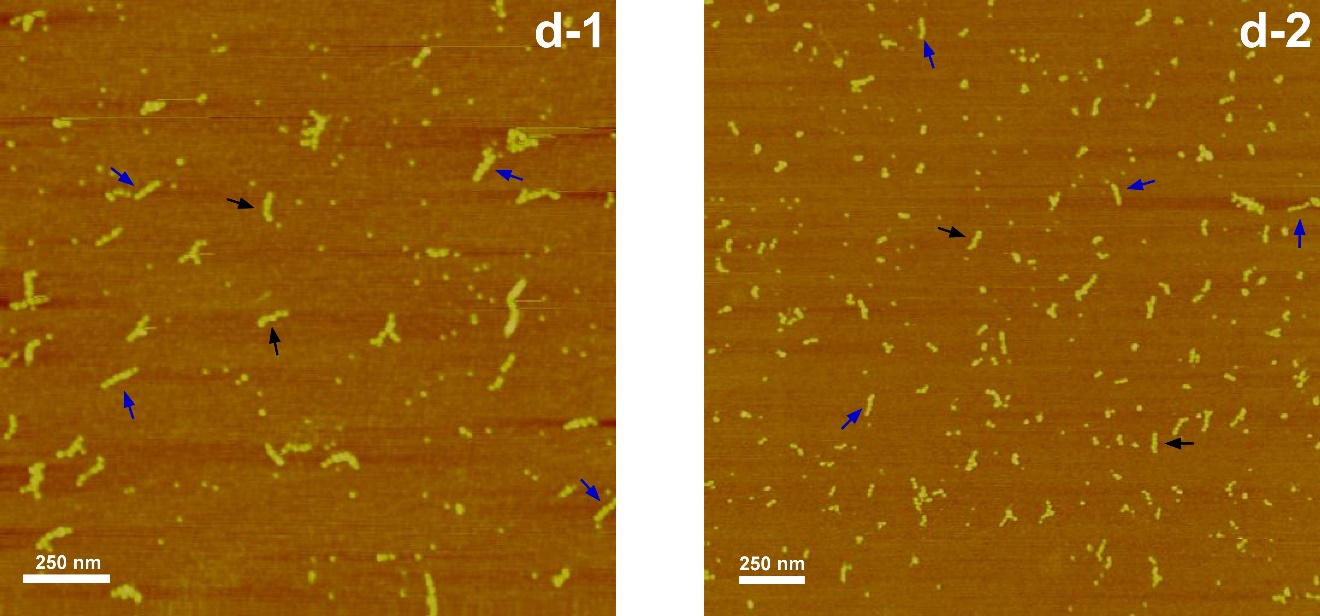
**

**
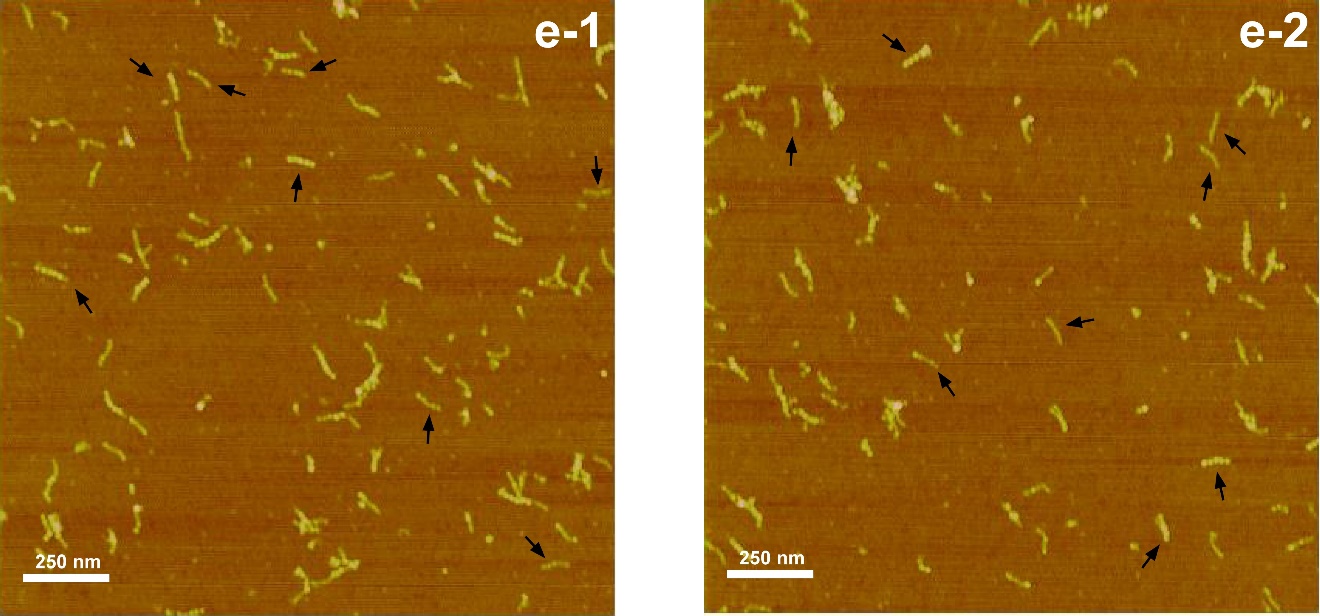
**

**
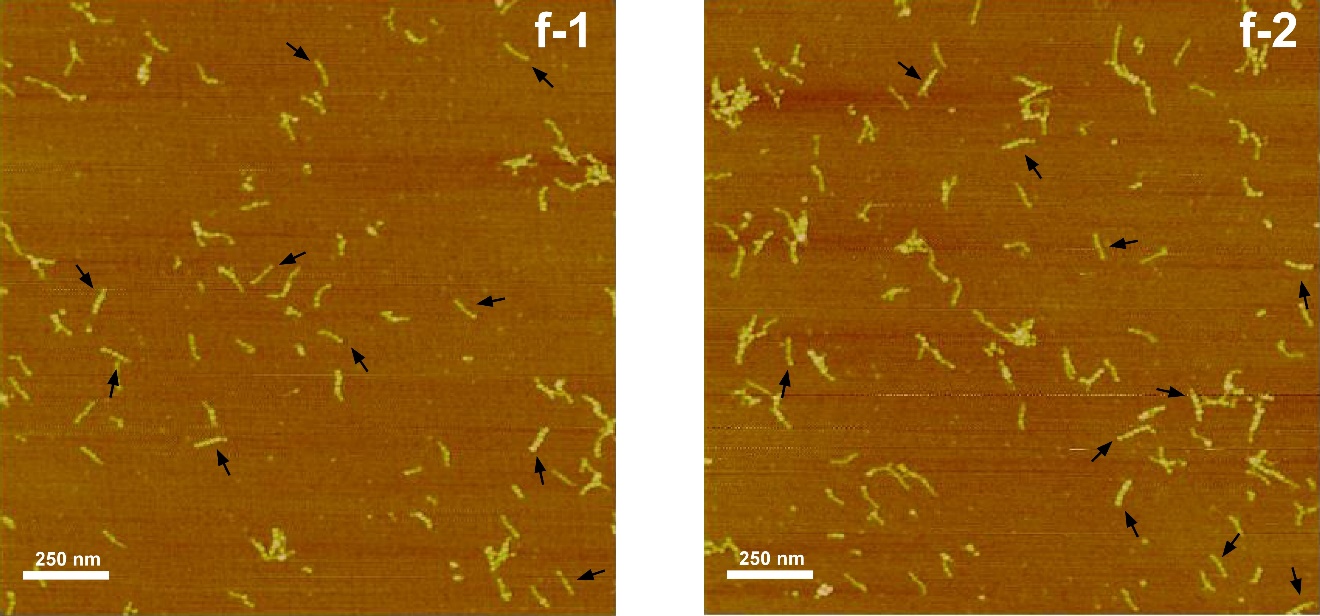
**

**
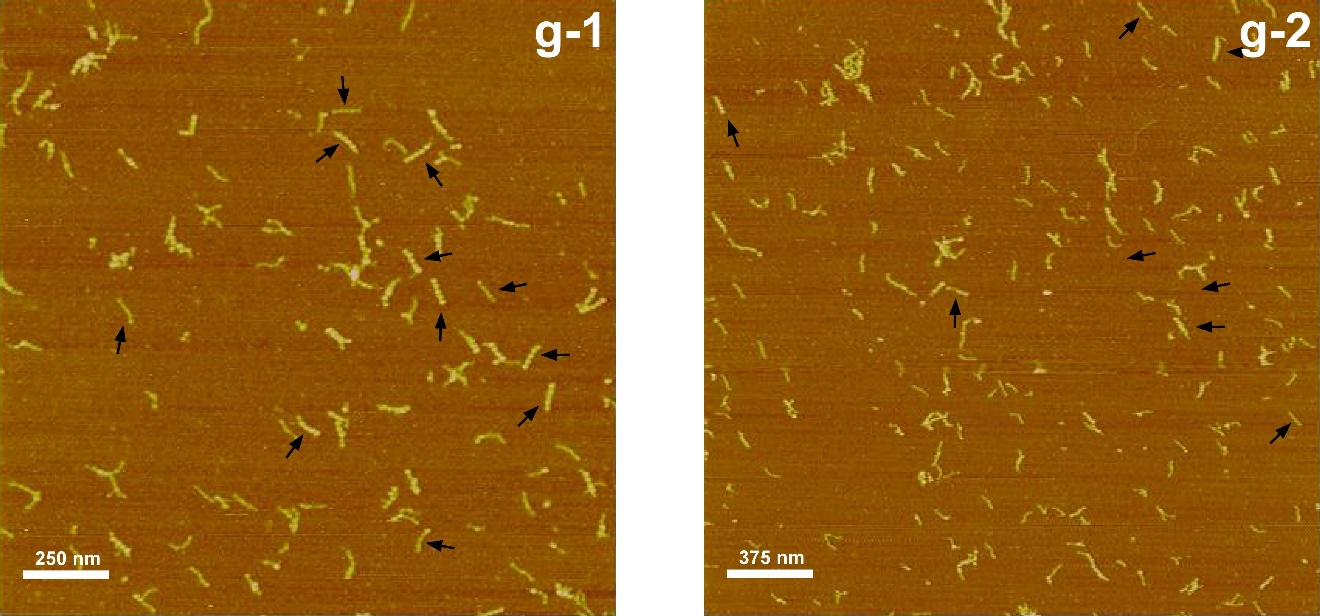
**

**
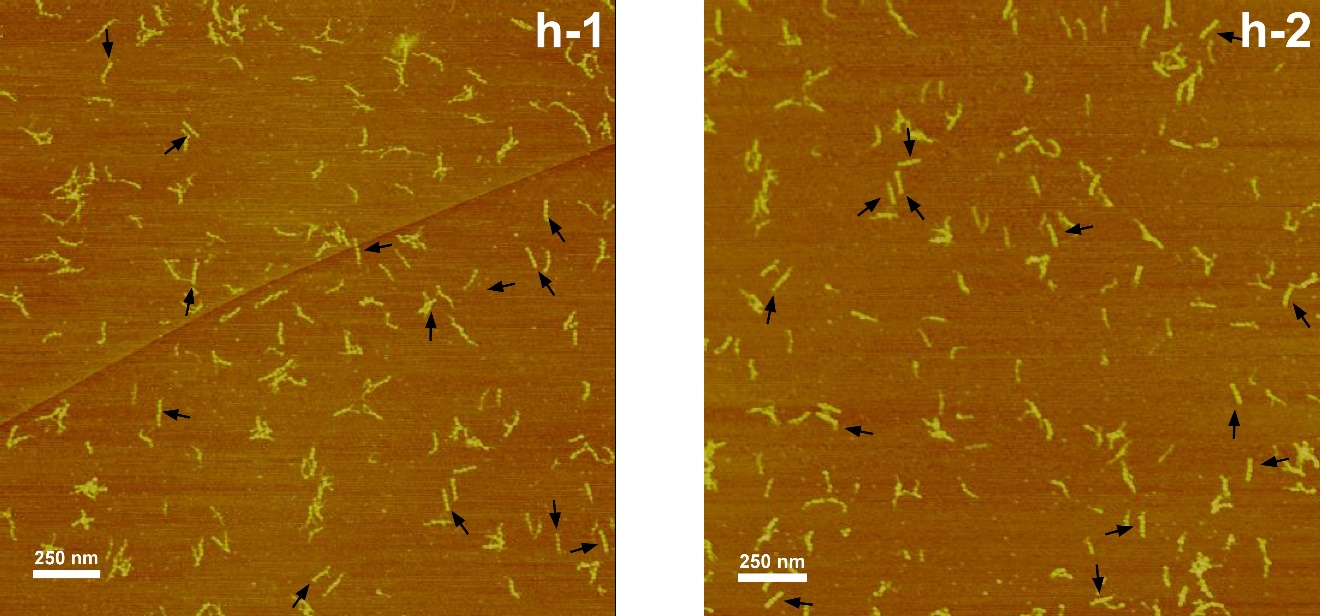
**

**
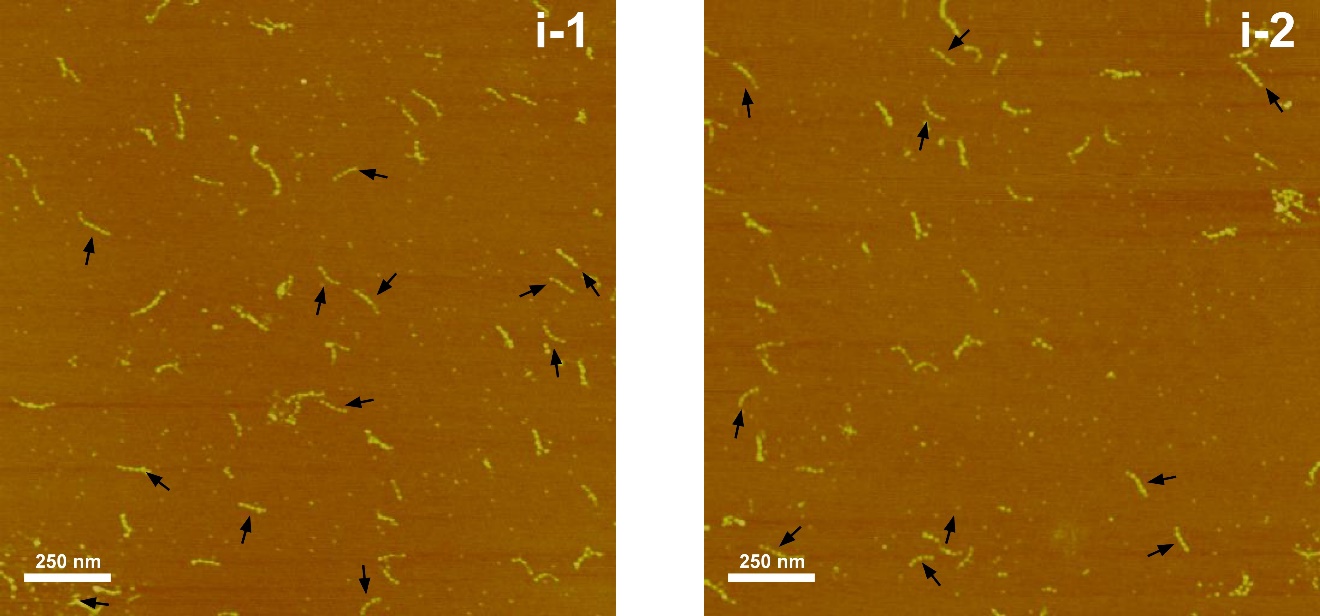
**

Figure S2. Selected AFM images for evaluating the frequency of occurrence for each variant: a, the linear labeled dsDNA; b, the linear labeled DX DNA; c, the zigzag labeled DX DNA; d, the zigzag labeled PX-DNA (5’, Δ=2); e, PX-DNA (5’, Δ=1); f, PX-DNA (3’, Δ=1); g, PX-DNA (3’, Δ=2); h, PX-DNA (5’, Δ=3); i, PX-DNA (3’, Δ=3).

**Table S1. Strand sequences of the DNA used**

(/phos/: 5’ phosphate, **T**: biotinylated thymine). Phosphorylated sites are positions where strands are ligated.

**Labeled DNA duplex:**

1. DS-11

GCTAATTGCTACCTCT**T**ATAAAGAAACTCGAATCGTCAGCGGGAAGCATTGCCGCTATTTCATTACCG**T**TATAGCCACGGTGCTGGCCGT

2. DS-12

/phos/TCGAAAGTTCTGATTGCTGACTCCAAGGGTC**T**CCACTTAGGCCACCTCTGGCATCGATCGGCCCATATATCCTGCTAACCCGA**T**CCCTTT

3. DS-13

/phos/ATCTGACTGCTGAATCTGCGTGCGTAACTATCGCTGGTCAAGTCTA**T**GTCACTGCCTAGCTCTAAAGGAATTTCCTAACGGTATCCGTAG

4. Linker-DS-112

AATCAGAACTTTCGAACGGCCAGCACCGTG

5. Linker-DS-123

ATTCAGCAGTCAGATAAAGGGATCGGGTTA

6. DS-21

CTACGGATACCGTTAGGAAATTCCTTTAGAGCTAGGCAGTGACATAGACTTGACCAGCGATAGTTACGCACGCAGATTCAGCAGTCAGAT

7. DS-22

/phos/AAAGGGATCGGGTTAGCAGGATATATGGGCCGATCGATGCCAGAGGTGGCCTAAGTGGAGACCCTTGGAGTCAGCAATCAGAACTTTCGA

8. DS-23

/phos/ACGGCCAGCACCGTGGCTATAACGGTAATGAAATAGCGGCAATGCTTCCCGCTGACGATTCGAGTTTCTTTATAAGAGGTAGCAATTAGC

9. Linker-DS-212

TAACCCGATCCCTTTATCTGACTGCTGAAT

10. Linker-DS-223

CACGGTGCTGGCCGTTCGAAAGTTCTGATT

**Labeled DX (1) (linear and zigzag):**

1. DX-11

GCTACGCCGTTGCTCTTCCAAAGAAACTCGAATCGTGTAGGGGAAGCATTGCCGCTATTTCATTACCGCGTGAGCCAAGGTGCTACCCGA

2. DX-12

/phos/GCGAAAGTTCTGATTGCTGACTCCAAGGGTGCCCACTTAGGCCACCTCTGGCATACATCGGCCCATCCATCCTGCTAACCCGATCCCTTG

3. DX-13

/phos/CCATGACTGCTGAATCTGCGTGCGTAACTATCGCTGGTCAAGTCTACGTCACTGCCTAGCTCTAGGAGAATTTCCTAACGGTATCCGTAG

4. Linker-DX-112

AATCAGAACTTTCGCTCGGGTAGCACCTTG

5. Linker-DX-123

5’-TAACAGCAGTCATGGCAAGGGATCGGGTTA

6. DX-21

TACTCATTGCTGAGTCCATGCTTACTTTGGCAATAACTCTGGGCTTTAATGATATGGTTTACTAAGTCTACTAGTTTCAACGATATAAAC

7. DX-22

/phos/GCCGGTCCTGACTTAGTGTAAAGTCCCCATGTACTGTACGGATGTCCCTATCATGTACCGGGTATTAGCGTAATGCTGTGTCAAATCTCT

8. DX-23

/phos/GTTAAACGTAGTACGAAGTTCTAAAGTAGATATAGTTATCTGTATTCGTAAGCTATCGTATCTAAAGAGTCATTCTACCGTACCATCTCT

9. Linker-DX-212

TAAGTCAGGACCGGCGTTTATATCGTTGAA

10. Linker-DX-223

CGTACTACGTTTAACAGAGATTTGACACAG

11. DX-4

CACGCGGTAATGAAATAGCGGCAATGCTTCCCCTACACGATTTACGATACGATACGAATACAGATAACTATATCTACTTTAGAA

12. DX-9

CTACGGATACCGTTAGGAAATTCTCCTAGACCAAAGTAAGCATGGACTCAGCAATGAGTA

13. DX-3

AGAGATGGTACGGTAGAATGACTCT**T**TAGACGAGTTTCTTTGGAAGAGCAACGGCGTAGC

14. DX-5 (Z)

GGAGTCAGCAATCAGAACTTTCGCTCGGGTAGCACC**T**TGGCTCTTCGTACTACGTTTAACAGAGATTTGACACAGCATTACGCT

15. DX-6

ATGGGCCGATGTATGCCAGAGGTGGCCTAAGTGGGGACCCTTAATACCCGGTACATGA**T**AGGGACATCCGTACAGTACATGGGG

16. DX-7 (Z)

CAGATTCAGCAGTCA**T**GGCAAGGGATCGGGTTAGCAGGATGGACTTTACACTAAGTCAGGACCGGCGTTTATATCGTTGAAACT

17. DX-8

GCTAGGCAGTGACGTAGACTTGACCAGCGATAGTTACGCACGAGTAGACTTAGTAAACCATATCATTAAAGCCCAGAGT**T**ATTG

18. DX-5 (L)

GGAGTCAGCAATCAGAACTTTCGCTCGGGTAGCACCTTGGCTCTTCG**T**ACTACGTTTAACAGAGATTTGACACAGCATTACGCT

19. DX-7 (L)

CAGATTCAGCAGTCATGGCAAGGGATCGGGTTAGCAGGATGGACTTTACACTAAGTCAGGACCGGCGT**T**TATATCGTTGAAACT

**Zigzag labeled PX (2):**

1. PXZ-11

GCTAATTGCTACCTCT**T**ATAAAGAAACTCGAATCGTCAGCGGGAAGCATTGCCGCTATTTCATTACCGCTA**T**AGCCACGGTGCTGGCCGT

2. PXZ-12

/phos/TCGAAAGTTCTGATTGCTGACTCCAAGGGTCCCCAC**T**TAGGCCACCTCTGGCATCGATCGGCCCATATATCCTGCTAACCCGATCCCTTT

3. PXZ-13

/phos/A**T**CTGACTGCTGAATCTGCGTGCGTAACTATCGCTGGTCAAGTCTACGTCACTGCC**T**AGCTCTAAAGGAATTTCCTAACGGTATCCGTAG

4. Linker-PXZ-112

AATCAGAACTTTCGAACGGCCAGCACCGTG

5. Linker-PXZ-123

ATTCAGCAGTCAGATAAAGGGATCGGGTTA

6. PXZ-21

CTACGGTTGATGTTAGGCGTCACCTTTATATTCAGGCAGTACGGTAGACTAAACCAGCGATCCTGACGCACGTACGGTCAGCAACCCTAT

7. PXZ-22

/phos/AAAGGGTCTGGGTTATGACTATATATGCTAAGATCGAATTGGGAGGTGCAAACAGTGGGCCAGATTGGAGAGGGAAATCAGTTTTATCGA

8. PXZ-23

/phos/ACCGTAAGCACCGTGGCAATAGCGTTACCGAAATATATACAATGCTCGGAACTGACGTGCGAAGTTTCCCAATAAGAGGTCGAAATTAGC

9. Linker-PXZ-212

TAACCCAGACCCTTTATAGGGTTGCTGACC

10. Linker-PXZ-223

CACGGTGCTTACGGTTCGATAAAACTGATT

11. PXZ-31

TTAGCATTCGAATCGCCATTGGCCCGACTCGCAGAGCTTTTCCGACGTGAGTATAGCGGGTGGTAACCCGTTTGCCATTTACCTTACGTC

12. PXZ-32

/phos/GACGTAAAAGCCCATTCCCTGCCGAATCTGGGACCAGGTTTGCGGTTTCCAATCAAAATTTAGCAAGCATAGTCAGCCAGTAGACCCCCG

13. PXZ-33

/phos/TGAGGGTGCTTAACCGTACAGTACTCAGGCAGGTCGGTTTCGGGTACCGTATGGATTGAATACGTTGATGACGTCCGTAATCAACGGTTA

14. Linker-PXZ-312

ATGGGCTTTTACGTCGACGTAAGGTAAATG

15. Linker-PXZ-323

GGTTAAGCACCCTCACGGGGGTCTACTGGC

16. PXZ-41

TAACCGATACCTACGGAAAATTTCAACGGAGCTAATCCATGACGTACCCGTGACCGACCTGAGTTAGTACTGCAGATTTAAGCGTCAGCA

17. PXZ-42

/phos/CGGGGGATCACTGGCGCAGGATGCTTGGGCCATTTTGTGCCAAAACCGGCCTACTGGTCGACCCTTCGGCTCAGCATGGGCAACTTCGTC

18. PXZ-43

/phos/GAGGCCAGGTAAATGGCTTTCGGGGTAATACCCGCGCGGCTCACGTTCCCGAAGCTCATTCGGTCGGGTTTATGGCGATTAGCATGCTAA

19. Linker-PXZ-412

GCCAGTGATCCCCCGTGCTGACGCTTAAAT

20. Linker-PXZ-423

CATTTACCTGGCCTCGACGAAGTTGCCCAT

**Titration with Δ=3:**

1. PXZ-11(33)

GCTAATTGCTACCTCTTAT**T**AAGAAACTCGAATCGTCAGCGGGAAGCATTGCCGCTATTTCATTACCGCTATAG**T**CACGGTGCTGGCCGT

2. PXZ-12(33)

/phos/TCGAAAGTTCTGATTGCTGACTCCAAGGGTCCCCACTTA**T**GCCACCTCTGGCATCGATCGGCCCATATATCCTGCTAACCCGATCCCTTT

3. PXZ-13(33)

/phos/ATCT**T**ACTGCTGAATCTGCGTGCGTAACTATCGCTGGTCAAGTCTACGTCACTGCCTAG**T**TCTAAAGGAATTTCCTAACGGTATCCGTAG

4. Linker-PXZ-112(33)

AATCAGAACTTTCGAACGGCCAGCACCGTG

5. Linker-PXZ-123(33)

ATTCAGCAGTAAGATAAAGGGATCGGGTTA

6. PXZ-41(33)

TAACCGATACCTACGGAAAATTTCAACGGAACTAATCCATGACGTACCCGTGACCGACCTGAGTTAGTACTGCAGATTTAAGCGTAAGCA

7. PXZ-42(33)

/phos/CGGGGGATCACTGGCGCAGGATGCTTGGGCCATTTTGTGCCAAAACCGGCATACTGGTCGACCCTTCGGCTCAGCATGGGCAACTTCGTC

8. PXZ-43(33)

/phos/GAGGCCAGGTAAATGACTTTCGGGGTAATACCCGCGCGGCTCACGTTCCCGAAGCTCATTCGGTCGGGTTAATGGCGATTAGCATGCTAA

9. Linker-PXZ-412(33)

GCCAGTGATCCCCCGTGCTTACGCTTAAAT

10. Linker-PXZ-423(33)

CATTTACCTGGCCTCGACGAAGTTGCCCAT

11. PXZ-11(35)

GCTAATTGCTACC**T**CTTATAAAGAAACTCGAATCGTCAGCGGGAAGCATTGCCGCTATTTCATTACCG**T**CATAGCCACGGTGCTGGCCGT

12. PXZ-12(35)

/phos/TCGAAAGTTCTGATTGCTGACTCCAAGGGTCCC**T**CCTTAGGCCACCTCTGGCATCGATCGGCCCATATATCCTGCTAACCCGATCCCT**T**T

13. PXZ-13(35)

/phos/ATCTGACTGCTGAATCTGCGTGCGTAACTATCGCTGGTCAAGTCTACGTCACT**T**GCTAGCTCTAAAGGAATTTCCTAACGGTATCCGTAG

14. Linker-PXZ-112(35)

AATCAGAACTTTCGAACGGCCAGCACCGTG

15. Linker-PXZ-123(35)

ATTCAGCAGTCAGATAAAGGGATCGGGTTA

16. PXZ-21(35)

CTACGGTTGATGTTAGGCGTCACCTTTATATTCAGCAAGTACGGTAGACTAAACCAGCGATCCTGACGCACGTACGGTCAGCAACCCTAT

17. PXZ-22(35)

/phos/AAAGGGTCTGGGTTATGACTATATATGCTAAGATCGAATTGGGAGGTGCAAACAGGAGGCCAGATTGGAGAGGGAAATCAGTTTTATCGA

18. PXZ-23(35)

/phos/ACCGTAAGCACCGTGGCAATGACGTTACCGAAATATATACAATGCTCGGAACTGACGTGCGAAGTTTCCCAATAAGAGGTCGAAATTAGC

19. Linker-PXZ-212(35)

5’-TAACCCAGACCCTTTATAGGGTTGCTGACC-3’

20. Linker-PXZ-223(35)

5’-CTCGGTGCTTACGGTTCGATAAAACTGATT-3’

**Titration with Δ=2:**

1. PXZ-11(23)

GCTAATTGCTACCTCTTA**T**AAAGAAACTCGAATCGTCAGCGGGAAGCATTGCCGCTATTTCATTACCGCTATA**T**CCACGGTGCTGGCCGT

2. PXZ-12(23)

/phos/TCGAAAGTTCTGATTGCTGACTCCAAGGGTCCCCACTT**T**GGCCACCTCTGGCATCGATCGGCCCATATATCCTGCTAACCCGATCCCTTT

3. PXZ-13(23)

/phos/ATC**T**GACTGCTGAATCTGCGTGCGTAACTATCGCTGGTCAAGTCTACGTCACTGCCTA**T**CTCTAAAGGAATTTCCTAACGGTATCCGTAG

4. Linker-PXZ-112(23)

AATCAGAACTTTCGAACGGCCAGCACCGTG

5. Linker-PXZ-123(23)

ATTCAGCAGTCAGATAAAGGGATCGGGTTA

6. PXZ-41(23)

TAACCGATACCTACGGAAAATTTCAACGGAGATAATCCATGACGTACCCGTGACCGACCTGAGTTAGTACTGCAGATTTAAGCGTCAGCA

7. PXZ-42(23)

/phos/CGGGGGATCACTGGCGCAGGATGCTTGGGCCATTTTGTGCCAAAACCGGCCAACTGGTCGACCCTTCGGCTCAGCATGGGCAACTTCGTC

8. PXZ-43(23)

/phos/GAGGCCAGGTAAATGGATTTCGGGGTAATACCCGCGCGGCTCACGTTCCCGAAGCTCATTCGGTCGGGTTTATGGCGATTAGCATGCTAA

9. Linker-PXZ-412(23)

GCCAGTGATCCCCCGTGCTGACGCTTAAAT

10. Linker-PXZ-423(23)

CATTTACCTGGCCTCGACGAAGTTGCCCAT

11. PXZ-11(25)

GCTAATTGCTACCT**T**TCATAAAGAAACTCGAATCGTCAGCGGGAAGCATTGCCGCTATTTCATTACCGC**T**ATAGCCACGGTGCTGGCCGT

12. PXZ-12(25)

/phos/TCGAAAGTTCTGATTGCTGACTCCAAGGGTCCCC**T**CTTAGGCCACCTCTGGCATCGATCGGCCCATATATCCTGCTAACC

13. PXZ-13(25)

/phos/CGATCCCTT**T**ATCTGACTGCTGAATCTGCGTGCGTAACTATCGCTGGTCAAGTCTACGTCACTG**T**CCAGCTCTAAAGGAATTTCCTAACGGTATCCGTAG

14. Linker-PXZ-112(25)

AATCAGAACTTTCGAACGGCCAGCACCGTG

15. Linker-PXZ-123(25)

CAGATAAAGGGATCGGGTTAGCAGGATATA

16. PXZ-21(25)

CTACGGTTGATGTTAGGCGTCACCTTTATATTCGGACAGTACGGTAGACTAAACCAGCGATCCTGACGCACGTACGGTCAGCAACCCTAT

17. PXZ-22(25)

/phos/AAAGGGTCTGGGTTATGACTATATATGCTAAGATCGAATTGGGAGGTGCAAACAGAGGGCCAGATTGGAGAGGGAAATCAGTTTTATCGA

18. PXZ-23(25)

/phos/ACCGTAAGCACCGTGGCAATAGCGTTACCGAAATATATACAATGCTCGGAACTGACGTGCGAAGTTTCCCAATGAAAGGTCGAAATTAGC

19. Linker-PXZ-212(25)

TAACCCAGACCCTTTATAGGGTTGCTGACC

20. Linker-PXZ-223(25)

CTCGGTGCTTACGGTTCGATAAAACTGATT

**Titration with Δ=1:**

1. PXZ-11(13)

GCTAATTGCTACCTCTT**T**TAAAGAAACTCGAATCGTCAGCGGGAAGCATTGCCGCTATTTCATTACCGCTAT**T**GCCACGGTGCTGGCCGT

2. PXZ-12(13)

/phos/TCGAAAGTTCTGATTGCTGACTCCAAGGGTCCCCACT**T**AGGCCACCTCTGGCATCGATCGGCCCATATATCCTGCTAACCCGATCCCTTT

3. PXZ-13(13)

/phos/AT**T**TGACTGCTGAATCTGCGTGCGTAACTATCGCTGGTCAAGTCTACGTCACTGCCT**T**GCTCTAAAGGAATTTCCTAACGGTATCCGTAG

4. Linker-PXZ-112(13)

AATCAGAACTTTCGAACGGCCAGCACCGTG

5. Linker-PXZ-123(13)

ATTCAGCAGTCAAATAAAGGGATCGGGTTA

6. PXZ-41(13)

TAACCGATACCTACGGAAAATTTCAACGGAGCAAATCCATGACGTACCCGTGACCGACCTGAGTTAGTACTGCAGATTTAAGCGTCAACA

7. PXZ-42(13)

/phos/CGGGGGATCACTGGCGCAGGATGCTTGGGCCATTTTGTGCCAAAACCGGCCTACTGGTCGACCCTTCGGCTCAGCATGGGCAACTTCGTC

8. PXZ-43(13)

/phos/GAGGCCAGGTAAATGGCATTCGGGGTAATACCCGCGCGGCTCACGTTCCCGAAGCTCATTCGGTCGGGTTTAAGGCGATTAGCATGCTAA

9. Linker-PXZ-412(13)

GCCAGTGATCCCCCGTGTTGACGCTTAAAT

10. Linker-PXZ-423(13)

CATTTACCTGGCCTCGACGAAGTTGCCCAT

11. PXZ-11(15)

GCTAATTGCTACCTC**T**TATAAAGAAACTCGAATCGTCAGCGGGAAGCATTGCCGCTATTTCATTACCGCT**T**TAGCCACGGTGCTGGCCGT

12. PXZ-12(15)

/phos/TCGAAAGTTCTGATTGCTGACTCCAAGGGTCCCCA**T**CTAGGCCACCTCTGGCATCGATCGGCCCATATATCCTGCTAACC

13. PXZ-13(15)

/phos/CGATCCCTTT**T**ACTGACTGCTGAATCTGCGTGCGTAACTATCGCTGGTCAAGTCTACGTCACTGC**T**CAGCTCTAAAGGAATTTCCTAACGGTATCCGTAG

14. Linker-PXZ-112(15)

AATCAGAACTTTCGAACGGCCAGCACCGTG

15. Linker-PXZ-123(15)

CAGTAAAAGGGATCGGGTTAGCAGGATATA

16. PXZ-21(15)

CTACGGTTGATGTTAGGCGTCACCTTTATATTCGAGCAGTACGGTAGACTAAACCAGCGATCCTGACGCACGTACGGTCAGCAACCCTTA

17. PXZ-22(15)

/phos/AAAGGGTCTGGGTTATGACTATATATGCTAAGATCGAATTGGGAGGTGCAAACGATGGGCCAGATTGGAGAGGGAAATCAGTTTTATCGA

18. PXZ-23(15)

/phos/ACCGTAAGCACCGTGGCAAAAGCGTTACCGAAATATATACAATGCTCGGAACTGACGTGCGAAGTTTCCCAATAAGAGGTCGAAATTAGC

19. Linker-PXZ-212(15)

TAACCCAGACCCTTTTAAGGGTTGCTGACC

20. Linker-PXZ-223(15)

CTCGGTGCTTACGGTTCGATAAAACTGATT

**Linear labeled PX (2) (44 bp):**

1. PXS-11

GCTAATTGCTACCTCT**T**ATAAAGAAACTCGAATCGTCAGCGGGAAGCATTGCCGCTATTC**T**ATTACCGCTATAGCCACGGTGCTGGCCGT

2. PXS-12

/phos/TCGAAAGTTCTGAT**T**GCTGACTCCAAGGGTCCCCACTTAGGCCACCTCTGGCATCGAC**T**GGCCCATATATCCTGCTAACCCGATCCCTTT

3. PXS-13

/phos/ATCTGACTGCTG**T**ATCTGCGTGCGTAACTATCGCTGGTCAAGTCTACGTCACTGCCTAGCTCTAAAGGAATTTCCTAACGGTATCCGTAG

4. Linker-PXS-112

AATCAGAACTTTCGAACGGCCAGCACCGTG

5. Linker-PXS-123

ATACAGCAGTCAGATAAAGGGATCGGGTTA

6. PXS-21

CTACGGTTGATGTTAGGCGTCACCTTTATATTCAGGCAGTACGGTAGACTAAACCAGCGATCCTGACGCACGTACGGACAGCAACCCTAT

7. PXS-22

/phos/AAAGGGTCTGGGTTATGACTATATATGCTAAAGTCGAATTGGGAGGTGCAAACAGTGGGCCAGATTGGAGAGGGAAATCAGTTTTATCGA

8. PXS-23

/phos/ACCGTAAGCACCGTGGCAATAGCGTTACCAGAATATATACAATGCTCGGAACTGACGTGCGAAGTTTCCCAATAAGAGGTCGAAATTAGC

9. Linker-PXS-212

TAACCCAGACCCTTTATAGGGTTGCTGTCC

10. Linker-PXS-223

CACGGTGCTTACGGTTCGATAAAACTGATT

**Linear labeled PX (2) (55 bp):**

1. PXS-55-11

GCTAATTGCTACCT**T**TCATAAAGAAACTCGAATCGTCAGCGGGAAGCATTGCCGCTATTTCATTACCGCTATAGCCACGGTGCTGGCCGT

2. PXS-55-13

/phos/CGATCCCTTTATCTGACTGCTGAATCTGCGTGCGTAACTATCGCTGGTCAAGTCTACGTCACTG**T**CCAGCTCTAAAGGAATTTCCTAACGGTATCCGTAG

3. PXS-55-31

TTAGCATTCGAATCGCCATTGGCCCGACTCGCAGAGCTTTTCCGACGTGAGTATAGCGGGTGGTAACCC**T**TTTGCCATTTACCTTACGTC

4. PXS-55-32

/phos/GACGTAAAAGCCCATTCCCTGCCGAATCTGGGACCAGGTTTGCGGTTTCCAATCAAAATTTAGCAAGCATAGTCAGCCAG

5. PXS-55-33

/phos/TAGACCCCC**T**TGAGGGTGCTTAACCGTACAGTACTCAGGCAGGTCGGTTTCGGGTACCGTATGGATTGAATACGTTGATGACGTCCGTAATCAACGGTTA

6. Linker-PXS-55-312

ATGGGCTTTTACGTCGACGTAAGGTAAATG

7. Linker-PXS-55-323

CCTCAAGGGGGTCTACTGGCTGACTATGCT

8. PXS-55-41

TAACCGATACCTACGGAAAATTTCAACGGAGCTAATCCATGACGTACCCGTGACCGACCTGAGTTAGTACTGCAGATTTAAGCGTCAGCA

9. PXS-55-42

/phos/AGGGGGATCACTGGCGCAGGATGCTTGGGCCATTTTGTGCCAAAACCGGCCTACTGGTCGACCCTTCGGCTCAGCATGGGCAACTTCGTC

10. PXS-55-43

/phos/GAGGCCAGGTAAATGGCTAAAGGGGTAATACCCGCGCGGCTCACGTTCCCGAAGCTCATTCGGTCGGGTTTATGGCGATTAGCATGCTAA

11. Linker-PXS-55-412

GCCAGTGATCCCCCTTGCTGACGCTTAAAT

12. Linker-PXS-55-423

CATTTACCTGGCCTCGACGAAGTTGCCCAT

**References**

1. Fu, T.J. & Seeman, N.C. (1993), DNA double-crossover molecules, Biochemistry 32: 3211-3220.

2. Shen, Z., Yan, H., Wang, T. and Seeman, N.C. (2004), Paranemic crossover DNA: A generalized Holliday structure with applications in nanotechnology, J. Am. Chem. Soc. 126: 1666-1674.
